# Valproic Acid-Induced Severe Acute Pancreatitis with Pseudocyst Formation: Report of a Case

**DOI:** 10.7759/cureus.297

**Published:** 2015-08-08

**Authors:** Sukanta Ray, Sujan Khamrui, Mohnish Kataria, Jayanta Biswas, Suman Saha

**Affiliations:** 1 Surgical Gastroenterology, School of Digestive and Liver Diseases, Institute of Postgraduate Medical Education and Research

**Keywords:** valproic acid, pancreatitis, pseudocyst, cystogastrostomy

## Abstract

Valproic acid is the most widely used anti-epilep­tic drug in children, and it is probably the most frequent cause of drug-induced acute pancreatitis. Outcomes for patients with valproic acid-associated pancreatitis vary from full recovery after discontinuation of the drug to severe acute pancreatitis and death. Here, we present a case of valproic acid-induced severe acute pancreatitis with pseudocyst formation in a 10-year-old girl with cerebral palsy and generalized tonic-clonic seizure. There was no resolution of the pseudocyst after discontinuation of valproic acid. The patient became symptomatic with a progressive increase in the size of the pseudocyst. She was successfully treated with cystogastrostomy and was well at 12-month follow-up.

## Introduction

Valproic acid (VPA) was approved in the United States in 1978 for the treatment of absence seizures. Since that time, it has been used either as monotherapy or in combination with other anticonvulsant agents for the treatment of mixed and complex partial seizures, acute manic episodes in bipolar disorder, and for prophylaxis of migraine headaches. Valproic acid is also effective in treating myoclonic, simple partial, and generalized tonic-clonic seizures [1]. Its mechanism is unknown; however, it is probably associated with the metabolism of the neurotransmitter GABA. In general, valproic acid offers advantages over older anticonvulsants in causing fewer troublesome adverse effects. It has a lower frequency of cognitive dysfunction and central nervous system effects, allowing patients to be more alert and functional. Common adverse effects include nausea and vomiting, tremor, and weight gain. The toxic effects it provokes can be dose-dependent or idiosyncratic. There are several VPA-re­lated idiosyncrasies, the most note­worthy being alopecia, bone marrow aplasia, im­mune-mediated hepatotoxicity, and pan­creatitis [2]. Fewer than 120 cases of VPA-related acute pancreatitis have been reported in the English literature. Most cases are mild and self-limiting. Herein, we report a case of VPA-related severe acute pancreatitis presented with large pseudocyst.

## Case presentation

**Figure 1:**
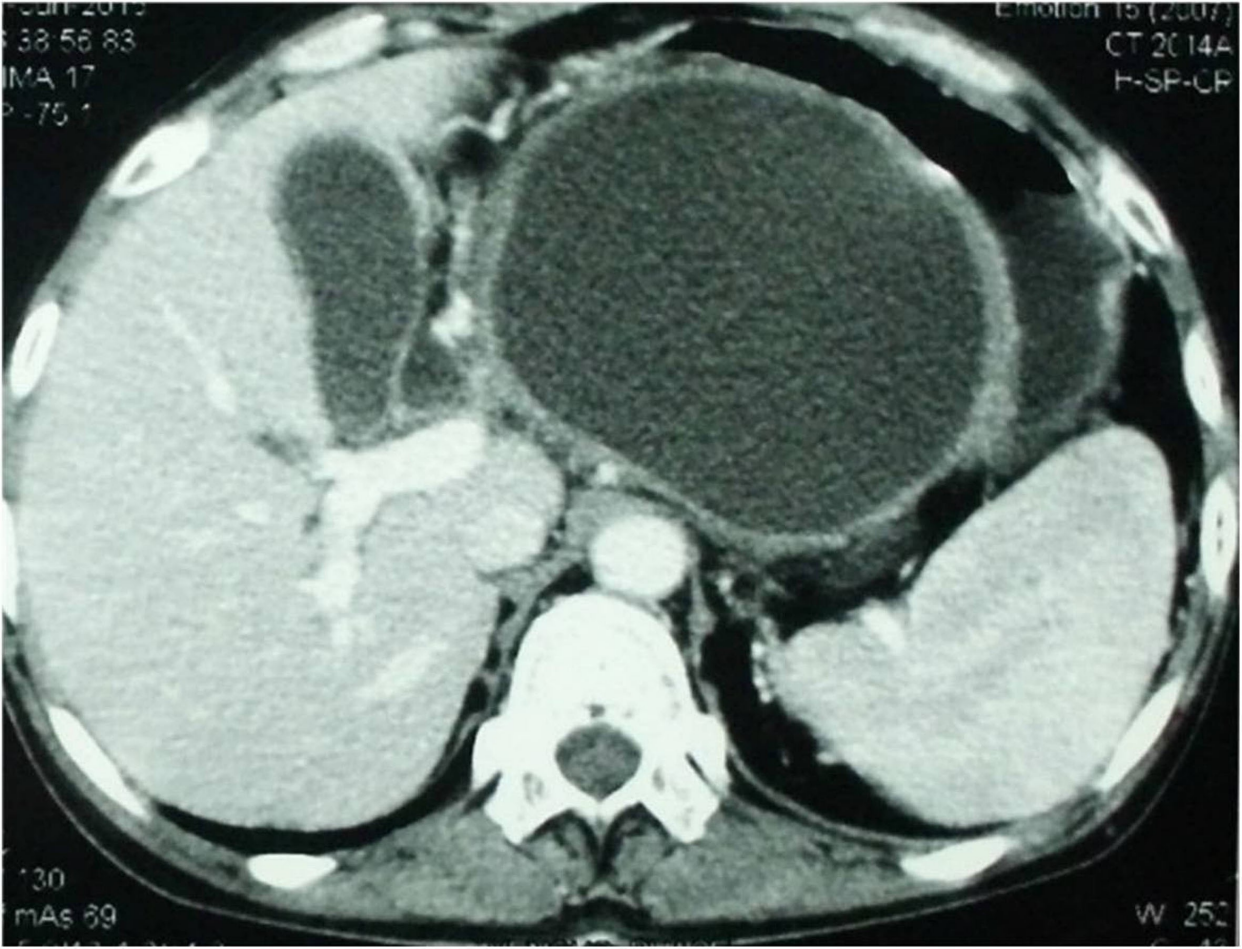
CT scan of the abdomen showing a large pseudocyst in the body and tail of the pancreas

A 10-year-old girl with cerebral palsy was admitted at our institution with recurrent episodes of abdominal pain radiating to the left side of the back associated with nausea and vomiting for over a period of eight months. Her physical examination revealed mild epigastric tenderness and a palpable lump in the left hypochondrium. Other system examinations were normal. She had a history of the first episode of abdominal pain eight months earlier when she was treated at a local hospital with conservative therapy and improved. Two months later, when she experienced a second episode of abdominal pain, she was evaluated with an abdominal ultrasound, which showed 7.2 x 5.7 cm cystic lesion in relation to the body and tail of the pancreas. There was no calculus in the gallbladder. An abdominal computed tomography (CT) scan at that time revealed a bulky pancreatic head with 5.5 x 6 cm cystic lesion in the tail of the pancreas. There were no internal septa, calcifications, or internal solid component. From her medical history, the attending physician came to know that she was taking valproic acid for generalized tonic-clonic seizure for about four years. She was not receiving any other drugs. Her serum amylase and lipase were significantly raised (361 U/L and 729 U/L, respectively). Viral serology for hepatitis A, B, C, cytomegalovirus, and herpes virus were negative. There was no evidence of hypertriglyceridemia and hypercalcemia. There was no history of trauma. After excluding other causes of pancreatitis, she was diagnosed as a case of valproic acid-induced pseudocyst of the pancreas. The Valproic acid was stopped, and she received symptomatic medical treatment and responded well. She was discharged from the hospital five days after admission with the advice of regular follow-up.  But, within 10 days of the withdrawal of the valproic acid, the seizures reappeared and levetiracetam was started. As there was no pseudocyst-related symptom and complication, she was on expectant management. Six months later, she was admitted at our institution with severe abdominal pain and vomiting. A CT scan of the abdomen at this admission revealed a large pseudocyst in the body and tail of the pancreas with intimate relation to the posterior wall of the stomach (Figure-1). We planned cystogastrostomy because of the increase in the size of the pseudocyst. As our gastroenterology colleagues have little experience in endoscopic cystogastrostomy in children and we have no expertise in laparoscopic cystogastrostomy, we performed open cystogastrostomy. Operative findings were a large pseudocyst in the body and tail of the pancreas closely adhered to the posterior wall of the stomach with left-sided portal hypertension. The patient had an uneventful recovery. The patient was doing well at her 12-month follow-up without any new episode of acute pancreatitis or seizure disorder.

Written informed consent was obtained from the parents of the patient for publication of the data.

## Discussion

Although numerous drugs have been listed in the etiology of acute pancreatitis (Table 1), drugs are a relatively uncommon cause of pancreatitis. Pancreatitis provoked by VPA is a rare entity, with an es­timated incidence of 1:40,000 [3], occurring mainly during the first year of treatment or after increase in the dose, with high­er incidence in young individuals, in poly­therapy (mainly with carbamazepine, phe­nytoin, phenobarbital, and some benzodi­azepines), with chronic encephalopathies, and in dialysis treatment [2-3].

**Table 1:** Drugs commonly associated with drug-induced pancreatitis.

Drug Class	Drug Name	Usual Onset Latency
Analgesics	Codeine	1 day
	Paracetamol	1 day
	Sulindac	"/ 30 days
Anesthetics	Propofol	1 day
Antidiabetics	Exenatide	
	Sitaglipin	
Anti-infectives		
Antivirals	Didanosine	"/ 30 days
	Lamivudine	
Antibacterials	Cotrimoxazole	1 – 30 days
	Erythromycin	1 day
	Tetracycline	
Antiparazitic agents	Pentamidine	1 – 30 days
	Stibogluconate	1 – 30 days
Anticonvulsants	Valproate	"/ 30 days
Antineoplastic agents	Asparaginase	1 – 30 days
	Cytarabine	1 – 30 days
Cardiovascular drugs		
ACE inhibitors	Enalapril	"/ 30 days
Diuretics	Furosemide	
Statins	Pravastatin	"/ 30 days
Gastrointestinal drugs	Mesalazine	1 – 30 days
	Omeprazole	"/ 30 days
Steroid hormones	Estrogens	"/ 30 days
	Glucocorticoids	1 – 30 days
Immunosuppressants	Azathioprine	1 – 30 days
	Sulfasalazine	1 – 30 days

Pancreatitis with valproic acid was first recognized in 1979 [4-5]. Since those initial reports, similar case reports have followed. Outcomes for patients with valproic acid-associated pancreatitis have ranged from full recovery after discontinuation of the drug to severe acute pancreatitis and death.

The mechanism by which valproic acid induces pancreatitis is unknown. However, it has been theorized that the depletion of the free radical scavengers, superoxide dismutase (SOD), catalase (CAT), and glutathione peroxidase occurs in patients receiving valproic acid [6-7]. Depletion of free radical scavengers could lead to the generation of excess free radicals, which in turn lead to endothelial permeability and lipid peroxidation, resulting in tissue damage. It has also been suggested that the reduction of carnitine brought about by the use of valproic acid has an important role in the damage caused to the pancreas [8].

Another proposed theory for valproic acid-induced pancreatitis involves the effect of valproic acid on mitochondrial b-oxidation. It has been established that valproic acid is eliminated primarily through mitochondrial b-oxidation, an enzyme system also involved in branched-chain amino acid metabolism. A research group demonstrated that valproic acid inhibited b-oxidation enzymes involved in branched-chain amino acid and straight-chain fatty acid metabolism [9]. They proposed that patients with a genetic deficiency in the enzymes involved in the mitochondrial b-oxidation of valproic acid may experience an increase in toxic metabolites. Another group screened serum and urine amino acid levels in patients who developed pancreatitis during valproic acid therapy [10].They did not observe a change in amino acid levels, and they discounted the b-oxidizing enzyme deficiency theory for valproic acid-induced pancreatitis.

The diagnosis of pancreatitis is made by means of the clinical signs and symptoms, among which stand out ab­dominal pain localized in the epigastrium, nausea that may be attended by vomiting, abdominal distension, fe­ver, and malaise [11]. The determination of blood amylase and lipase levels is fundamental, as their increase helps to orient us and may confirm the clinical suspicion. Am­ylase levels may increase in some patients receiving val­proic acid without turning into pancreatitis [12]. In a patient receiving valproic acid, a diagnosis of pancreatitis must be established with increased levels of pancreatic enzymes together with the presence of clinical symptoms. The in­crease of lipase, which is secreted only by the pancreas, is a more specific indication of pancreatic damage. Depending upon the clinical scenario, further investigations like abdominal ultrasound and CT scan may be required. An ultrasound is useful in the preliminary evaluation of patients suspected of having acute pancreatitis, and it must be done within the first 24 to 48 hours from the on­set of the clinical symptoms. Thus, ultrasonography of the pancreatic duct is valuable in diagnosis and monitoring of pancreatitis in children [13]. An abdominal CT scan must be performed on every pa­tient with severe acute pancreatitis, as it plays an im­portant role to assess the extent of the inflammatory process, the presence of ne­crosis, and other local complications.

VPA-related acute pancreatitis is basically a diagnosis of exclusion and should be considered when other reasonable causes of pancreatitis are not present. In our case, all other possible causes of pancreatitis were ruled out. There was no evidence of gallstones. Serum values of calcium and triglycerides were normal. There was no family history of pancreatitis and the patient was not receiving any other medication except valproic acid.

As most cases are mild and self-limiting, the withdrawal of valproic acid may permit normal­ization of the amylase levels and the disappearance of the clinical symptomatology [12]. However, in our case, the patient remained symptomatic as the patient had a severe episode of pancreatitis with pseudocyst formation. The use of valproic acid must be avoided in patients who have had acute pancreatitis associated with the use of valp­roic acid, due to its high relapse rate and complications [4, 14].

## Conclusions

Acute pancreatitis must always be con­sidered when a child receiving treatment with valproic acid shows gastrointestinal symptoms, as it is a complication that may be difficult to diagnose. Raised pancreatic amylase and lipase would confirm the clinical suspicion. A CT scan will help in the assessment of the local complications. The suspension of the administration of valproic acid in patients who have acute pancreatitis is mandatory, and the drug must not be resumed once the patient has recovered.
